# A New Discrete Analogue of the Continuous Muth Distribution for Over-Dispersed Data: Properties, Estimation Techniques, and Application

**DOI:** 10.3390/e27040409

**Published:** 2025-04-10

**Authors:** Howaida Elsayed, Mohamed Hussein

**Affiliations:** 1 Department of Business Administration, College of Business, King Khalid University, Abha 61421, Saudi Arabia; m.hussein@alexu.edu.eg; 2 Department of Mathematics and Computer Science, Alexandria University, Alexandria 21544, Egypt

**Keywords:** muth distribution, discretization approach, reliability analysis, estimation techniques, Monte Carlo simulation, 60E05, 62F10, 62E10, 62N05

## Abstract

We present a new one-parameter discrete Muth (DsMuth) distribution, a flexible probability mass function designed for modeling count data, particularly over-dispersed data. The proposed distribution is derived through the survival discretization approach. Several of the proposed distribution’s characteristics and reliability measures are investigated, including the mean, variance, skewness, kurtosis, probability-generating function, moments, moment-generating function, mean residual life, quantile function, and entropy. Different estimation approaches, including maximum likelihood, moments, and proportion, are explored to identify unknown distribution parameters. The performance of these estimators is assessed through simulations under different parameter settings and sample sizes. Additionally, a real dataset is used to emphasize the significance of the proposed distribution compared to other available discrete probability distributions.

## 1. Introduction

Modeling count data plays an essential role in various fields, including medicine, public health, sociology, medicine, agriculture, epidemiology, and applied science. Various probability distributions have been established to model count data, particularly count data with over-dispersion. Nonetheless, we need to become more adaptable in terms of discrete distributions to effectively model count data sets with high over-dispersion. The Poisson distribution is a widely used model for count data. It is well known that in the Poisson distribution, the mean and variance are equal. This characteristic of the Poisson distribution poses challenges in modeling actual life datasets [1]. In practical applications, count data is often over-dispersed, meaning that the empirical variance exceeds the empirical mean. In this case, using the Poisson distribution for these types of data sets yields misspecification of the underlying probability distribution. This occurs because real-life count data often exhibit either over-dispersion, where the variance is greater than the mean, or under-dispersion, where the variance is lower than the mean. Negative binomial distribution is commonly used as the primary choice for modeling over-dispersed count data. Consequently, several discrete distributions have been developed based on well-known continuous models used in reliability analysis, failure time studies, and related fields.

Discretizing continuous distributions can be achieved through various approaches, including survival discretization, mixed-Poisson models, and infinite series methods. Among these, the survival discretization approach is the most commonly used [2]. The discretization of continuous probability distributions has attracted considerable attention in recent years. Various discrete distributions have been proposed and analyzed in the literature based on the discretization of the survival function. For instance, various discrete distributions have been introduced and studied, including the discrete Rayleigh [3], discrete half-normal [4], discrete Burr and Pareto [5], discrete exponential [6], discrete inverse-Weibull [7], discrete Power Ailamujia [8], discrete Rayleigh generator [9], new generalization of the geometric [10], discrete Lindley [11], discrete Burr-XII [12], discrete Burr III [13], two-parameter discrete Lindley [14], discrete log-logistic [15], new discrete distribution [16], Poisson Ailamujia [17], discrete alpha power inverse Lomax [18], Uniform Poisson and Ailamujia [19], discrete Half-Logistic [20], discrete Marshall-Olkin Weibull [21], discrete Gompertz-G family [22], Discrete Bilal [23], discrete Burr-Hatke [24], three-parameter discrete Lindley [25], new discrete Lindley [26], exponentiated discrete Lindley [27], discrete generalized Lindley [28], discrete Gumble [29], discrete inverted Topp-Leone [30], discrete Ramos-Louzada distribution [31], generalized exponential type II [32], and discrete inverse-Rayleigh [33] distributions.

Although numerous distributions exist in the literature for analyzing lifetime data, there remains a need for more flexible and adaptable distributions that can effectively model data under different conditions. The continuous Muth distribution, introduced by [34], is a one-parameter lifetime distribution that has proven useful in modeling various reliability phenomena. It may be valuable to explore the development of a discrete version of the Muth distribution, enabling its implementation in modeling discrete data. An essential purpose of this paper is to propose a new discrete analog based on the continuous Muth distribution, referred to as the discrete Muth (DsMuth) distribution. As a fundamental mathematical definition, the proposed distribution is characterized by the subsequent probability density function (PDF) and cumulative distribution function (CDF), respectively.

(1) $$\begin{array}{cc} f(x;\alpha ) & =\left(e^{\alpha x}-\alpha \right)e^{\alpha x-\frac{1}{\alpha }\left(e^{\alpha x}-1\right)},\;x\geq 0\;\mathrm{and}\;\alpha \in \left(0,1\right] \end{array}$$

(2) $$\begin{array}{cc} F(x;\alpha ) & =P\left(X<x\right)=1-e^{\alpha x-\frac{1}{\alpha }\left(e^{\alpha x}-1\right)},\;x>0\;\mathrm{and}\;\alpha \in \left(0,1\right] \end{array}$$

The corresponding survival function (SF) and hazard rate function (HRF) related to Equation (2) can be formulated as:

(3) $$\begin{array}{cc} S(x;\alpha ) & =P\left(X\geq x\right)=e^{\alpha x-\frac{1}{\alpha }\left(e^{\alpha x}-1\right)} \end{array}$$

(4) $$\begin{array}{cc} h(x;\alpha ) & =\frac{\left(e^{\alpha x}-\alpha \right)e^{\alpha x-\frac{1}{\alpha }\left(e^{\alpha x}-1\right)}}{e^{\alpha x-\frac{1}{\alpha }\left(e^{\alpha x}-1\right)}} \end{array}$$

In this article, we propose a discrete from of the Muth distribution using the survival discretization method, referred to as the discrete Muth distribution. The DsMuth distribution is a feasible alternative for modeling the over-dispersed count data set. A comprehensive study of the statistical characteristics of the DsMuth distribution is conducted. Additionally, different simulation studies are presented to estimate the unknown parameter of the DsMuth distribution and study the behavior of the maximum likelihood estimation (MLE), moments estimation (MOM), and proportion estimation (PE) methods. The suggested distribution demonstrates superior effectiveness in modeling over-dispersed count data compared to existing competing distributions.

The article is structured as outlined below: Section 2 is dedicated to deriving the discrete version of the Muth distribution, accompanied by graphical representations of its probability mass function (PMF) and hazard rate function (HRF). Section 3 presents the derivation of various mathematical characteristics of the proposed distribution. Section 4 introduces the DsMuth distribution’s entropy, which measures its uncertainty and randomness. Section 5 introduces quantiles of a discrete random variable. Section 6 presents the parameter estimation employing the maximum likelihood method, the method of moments, and the proportion estimation method. Additionally, Section 7 presents simulation experiments to assess the effectiveness of the estimation methods under various sample sizes and parameter values. Section 8 utilizes a real dataset to validate the applicability of the DsMuth distribution in modeling count data and to demonstrate the flexibility of the proposed distribution. Finally, Section 9 provides a summary of the results and presents the conclusions.

## 2. The DsMuth Distribution

Let *X* be a continuous random variable with survival function S(x)=P(X≥x). Using the survival discretization approach, we define a discrete random variable Y=⌊X⌋ (i.e., the greatest integer less than or equal to *X*). Then, the probability mass function (pmf) of the discrete random variable *Y* follows as:

(5) $$\begin{array}{cc} P(y) & =P(y\leq X<y+1)\\ \; & =P(X\geq y)-P(X\geq y+1)\\ \; & =S(y)-S(y+1),\;y=0,1,2,\ldots \end{array}$$

By employing the survival discretization approach in Equation (5), a random variable *X* is said to follow the DsMuth distribution. If the survival function of the Muth distribution with parameter $\lambda =e^{\alpha }$ is expressed as:

(6) $$S\left(x;\lambda \right)=\lambda^{x}\exp\left\{-\frac{1}{\ln\lambda }\left(\lambda^{x}-1\right)\right\},\;x=0,1,2,\ldots$$

The corresponding probability mass function (pmf) and the cumulative distribution function (CDF) can be represented as:

$$\begin{array}{cc} p(x;\alpha ) & =e^{\alpha x-\frac{1}{\alpha }\left(e^{\alpha x}-1\right)}-e^{\alpha \left(x+1\right)-\frac{1}{\alpha }\left(e^{\alpha (x+1)}-1\right)},\;x=0,1,2,\ldots \end{array}$$

By performing the variable transformation $\lambda =e^{\alpha }$ (i.e., α=lnλ), we obtain

(7) $$\begin{array}{cc} p(x) & =\lambda^{x}\exp\left\{-\frac{1}{\ln\lambda }\left(\lambda^{x}-1\right)\right\}-\lambda^{x+1}\exp\left\{-\frac{1}{\ln\lambda }\left(\lambda^{x+1}-1\right)\right\},\;x=0,1,2,\ldots \end{array}$$

The cumulative distribution function (CDF) of the DsMuth distribution is defined based on the survival function as follows:

(8) $$\begin{array}{ll} F(x;\lambda ) & =P\left(X\leq x;\lambda \right)=1-S\left(x;\lambda \right)+P\left(X=x;\lambda \right)=1-\left[e^{\alpha \left(x+1\right)-\frac{1}{\alpha }\left(e^{\alpha (x+1)}-1\right)}\right]\\ F(x;\lambda ) & =1-\lambda^{x+1}\exp\left\{-\frac{1}{\ln\lambda }\left(\lambda^{x+1}-1\right)\right\},\;x=0,1,2,\ldots \end{array}$$

where $\lambda =e^{\alpha }$, 1<λ≤e, and x=0,1,2,3,…

The survival function (SF) of the DsMuth model can be written as

(9) $$\begin{array}{c} S\left(x;\alpha \right)=\lambda^{x+1}\exp\left\{-\frac{1}{\ln\lambda }\left(\lambda^{x+1}-1\right)\right\}. \end{array}$$

**Figure 1:**
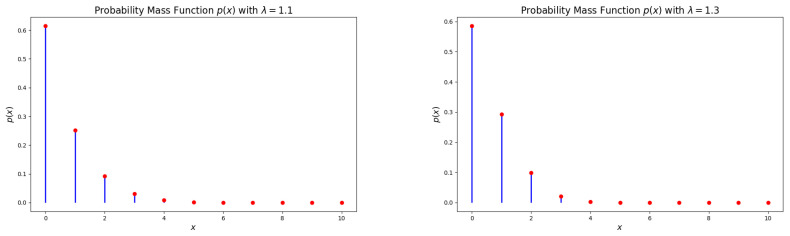
Graphical representations of the probability mass function (pmf) for the discrete Muth (DsMuth) distribution: λ=1.1 (**left**) and λ=1.3 (**right**). 1. Red dots represent the actual values of p(x) at specific points. 2. Blue line represents the probability distribution showing how the probability changes with different values of x.

**Figure 2:**
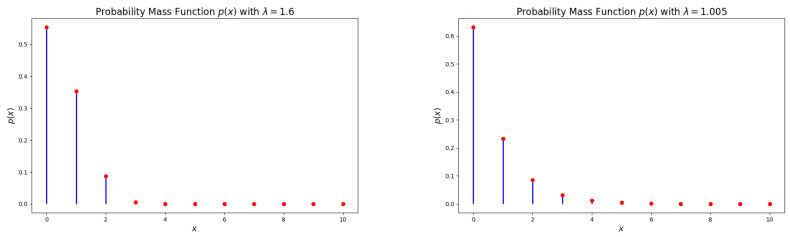
Graphical representations of the probability mass function (pmf) for the discrete Muth (DsMuth) distribution: λ=1.6 (**left**) and λ=1.005 (**right**). 1. Red dots represent the actual values of p(x) at specific points. 2. Blue line represents the probability distribution showing how the probability changes with different values of x.

**Figure 3:**
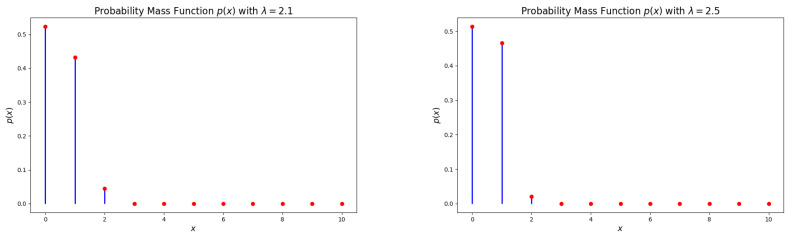
Graphical representations of the probability mass function (pmf) for the discrete Muth (DsMuth) distribution: λ=2.1 (**left**) and λ=2.5 (**right**). 1. Red dots represent the actual values of p(x) at specific points. 2. Blue line represents the probability distribution showing how the probability changes with different values of x.

The probability mass function (PMF) of the DsMuth distribution, as stated in Equation (7), is log-concave for all values of λ∈(1,e]. Figure 1, Figure 2 and Figure 3 illustrate various possible shapes of the PMF of the DsMuth distribution for various values of the parameter λ. It has been noted that the PMF of DsMuth is suitable for modeling positively skewed data with a unimodal shape for any value of λ.

The DsMuth distribution’s hazard rate function (HRF), reversed hazard rate (RH), and second failure rate ($r^{*}h$) can be formulated as follows:

(10) $$\begin{array}{cc} h(x;\alpha ) & =\frac{p(x;\alpha )}{1-F(x-1;\alpha )}=1-\frac{\lambda^{\left(x+1\right)}\exp\left\{-\frac{1}{\ln\lambda }\left(\lambda^{\left(x+1\right)}-1\right)\right\}}{\lambda^{x}\exp\left\{-\frac{1}{\ln\lambda }\left(\lambda^{x}-1\right)\right\}} \end{array}$$

(11) $$\begin{array}{cc} rh(x;\alpha ) & =\frac{p(x;\alpha )}{F(x;\alpha )}=\frac{\lambda^{x}\exp\left\{-\frac{1}{\ln\lambda }\left(\lambda^{x}-1\right)\right\}-\lambda^{\left(x+1\right)}\exp\left\{-\frac{1}{\ln\lambda }\left(\lambda^{\left(x+1\right)}-1\right)\right\}}{1-\lambda^{\left(x+1\right)}\exp\left\{-\frac{1}{\ln\lambda }\left(\lambda^{\left(x+1\right)}-1\right)\right\}} \end{array}$$

(12) $$\begin{array}{cc} r^{*}h\left(x;\alpha \right) & =\log\left[\frac{S(x;\alpha )}{S(x+1;\alpha )}\right]=\log\left[\frac{\lambda^{\left(x+1\right)}\exp\left\{-\frac{1}{\ln\lambda }\left(\lambda^{\left(x+1\right)}-1\right)\right\}}{\lambda^{\left(x+2\right)}\exp\left\{-\frac{1}{\ln\lambda }\left(\lambda^{\left(x+2\right)}-1\right)\right\}}\right],\;x=0,1,2,\cdots \end{array}$$

**Figure 4:**
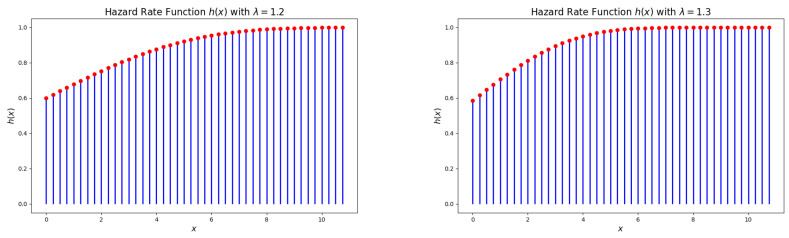
Plots of the hazard rate function (HRF) for the DsMuth distribution: λ=1.2 (**left**) and λ=1.3 (**right**). 1. Red dots represent the actual values of p(x) at specific points. 2. Blue line represents the probability distribution showing how the probability changes with different values of x.

**Figure 5:**
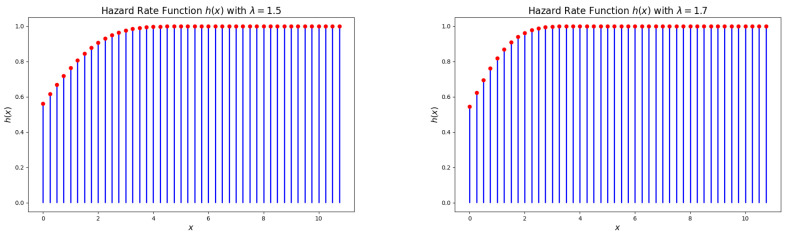
Plots of the hazard rate function (HRF) for the DsMuth distribution: λ=1.5 (**left**) and λ=1.7 (**right**). 1. Red dots represent the actual values of p(x) at specific points. 2. Blue line represents the probability distribution showing how the probability changes with different values of x.

Figure 4 and Figure 5 illustrate that the hazard rate function (HRF) of the DsMuth distribution is consistently increasing with respect to *x* for all values of the parameter λ.

## 3. Some Properties of the DsMuth Distribution

In this section, the probability generating function (pgf), the rth moment, and the quantile function of the DsMuth distribution are examined.

### 3.1. Probability Generating Function (PGF)

Let *X* be a discrete random variable defined on the set of non-negative integers {0,1,2,⋯}. The probability generating function $G_{X}\left(s\right)$ of the DsMuth distribution is obtained as:

(13) $$\begin{array}{cc} G_{X}\left(s\right) & =E\left(s^{X}\right)=\sum_{x=0}^{\infty }s^{x}P\left(X=x\right)=\sum_{x=0}^{\infty }s^{x}\left[S\left(x\right)-S\left(x+1\right)\right].\\ G_{X}\left(s\right) & =1+\left(s-1\right)\sum_{x=1}^{\infty }s^{\left(x-1\right)}S\left(x\right)\\ G_{X}\left(s\right) & =1+\left(s-1\right)\sum_{x=1}^{\infty }s^{\left(x-1\right)}\lambda^{x}\exp\left(-\frac{1}{\ln\lambda }\left(\lambda^{x}-1\right)\right). \end{array}$$

The factorial moments can be derived from the probability generating function (PGF) of the DsMuth distribution. To find the factorial moments using the PGF, we use the relationship between the derivatives of the PGF and the factorial moments. Specifically, the *n*-th factorial moment $\mu_{n}^{\prime }$ is given by:

$$\mu_{n}^{\prime }={\left.\frac{d^{n}}{ds^{n}}G_{X}\left(s\right)\right|}_{s=1}$$

The first four factorial moments, denoted as $\left(\mu_{1}^{\prime },\mu_{2}^{\prime },\mu_{3}^{\prime },\mu_{4}^{\prime }\right)$, can be determined utilizing the factorial moments as:

First factorial moment of the DsMuth Distribution

The first factorial moment (mean) of the DsMuth distribution is obtained by differentiating the probability generating function $G_{X}\left(s\right)$ with respect to *s* and then setting s=1:

(14) $$\begin{array}{cc} E[X] & =G_{X}^{\prime }\left(s\right){|}_{s=1}=\frac{d}{ds}G_{X}\left(s\right){|}_{s=1}.\\ G_{X}^{\prime }\left(s\right) & =\sum_{x=1}^{\infty }s^{\left(x-1\right)}\lambda^{x}\exp\left(-\frac{1}{\ln\lambda }\left(\lambda^{x}-1\right)\right)\\ \; & \;+\left(s-1\right)\sum_{x=1}^{\infty }\left(x-1\right)s^{\left(x-2\right)}\lambda^{x}\exp\left(-\frac{1}{\ln\lambda }\left(\lambda^{x}-1\right)\right)\\ G_{X}^{\prime }\left(1\right) & =E\left[X\right]=\sum_{x=1}^{\infty }\lambda^{x}\exp\left(-\frac{1}{\ln\lambda }\left(\lambda^{x}-1\right)\right) \end{array}$$

Second factorial moment: Differentiating $G_{X}^{\prime }\left(s\right)$ once more with respect to and setting s=1, we obtain

(15) $$\begin{array}{cc} G_{X}^{\prime \prime }\left(s\right) & =\sum_{x=1}^{\infty }\left(x-1\right)s^{\left(x-2\right)}\lambda^{x}\exp\left(-\frac{1}{\ln\lambda }\left(\lambda^{x}-1\right)\right)\\ \; & \;+\sum_{x=1}^{\infty }\left(x-1\right)s^{\left(x-2\right)}\lambda^{x}\exp\left(-\frac{1}{\ln\lambda }\left(\lambda^{x}-1\right)\right)\\ \; & \;+\left(s-1\right)\sum_{x=1}^{\infty }\left(x-1\right)\left(x-2\right)s^{\left(x-3\right)}\lambda^{x}\exp\left(-\frac{1}{\ln\lambda }\left(\lambda^{x}-1\right)\right)\\ G_{X}^{\prime \prime }\left(1\right) & =\mu_{2}^{\prime }=2\sum_{x=1}^{\infty }\left(x-1\right)\lambda^{x}\exp\left(-\frac{1}{\ln\lambda }\left(\lambda^{x}-1\right)\right). \end{array}$$

Third factorial moment: Differentiating $G_{X}^{\prime \prime }\left(s\right)$ once more with respect to and setting s=1, we have

(16) $$\begin{array}{cc} G_{X}^{\prime \prime \prime }\left(1\right) & =\mu_{3}^{\prime }=3\sum_{x=1}^{\infty }\left(x-1\right)\left(x-2\right)\lambda^{x}\exp\left(-\frac{1}{\ln\lambda }\left(\lambda^{x}-1\right)\right). \end{array}$$

Fourth factorial moment: Differentiating $G_{X}^{\prime \prime \prime }\left(s\right)$ once more with respect to and putting s=1, we obtain

(17) $$\begin{array}{cc} G_{X}^{\prime \prime \prime \prime }\left(1\right) & =\mu_{4}^{\prime }=4\sum_{x=1}^{\infty }\left(x-1\right)\left(x-2\right)\left(x-3\right)\lambda^{x}\exp\left(-\frac{1}{\ln\lambda }\left(\lambda^{x}-1\right)\right). \end{array}$$

By substituting *s* by $e^{s}$,( i.e., $s=e^{s}$), in Equation (13), the moment generating function (MGF) can be expressed as:

(18) $$\begin{array}{cc} M_{X}\left(e^{s}\right) & =1+\left(e^{s}-1\right)\sum_{x=1}^{\infty }{\left(e^{s}\right)}^{\left(x-1\right)}\lambda^{x}\exp\left(-\frac{1}{\ln\lambda }\left(\lambda^{x}-1\right)\right). \end{array}$$

The first four moments around the origin of the DsMuth distribution can be computed using the moment generating function as follows:

Differentiating the moment generating function (MGF) $M_{X}\left(e^{s}\right)$ with respect to *s* and setting s=0, we obtain the mean of the DsMuth distribution:

$$\begin{array}{cc} E(X) & =\frac{dM_{X}\left(e^{s}\right)}{ds}=e^{s}\sum_{x=1}^{\infty }{\left(e^{s}\right)}^{\left(x-1\right)}\lambda^{x}\exp\left(-\frac{1}{\ln\lambda }\left(\lambda^{x}-1\right)\right)\\ \; & \;+\left(e^{s}-1\right)\sum_{x=1}^{\infty }\left(x-1\right){\left(e^{s}\right)}^{\left(x-1\right)}\lambda^{x}\exp\left(-\frac{1}{\ln\lambda }\left(\lambda^{x}-1\right)\right). \end{array}$$

By setting s=0, we obtain the expected value:

$$\begin{array}{cc} E(X) & ={\left.\frac{dM_{X}\left(e^{s}\right)}{ds}\right|}_{s=0}=\sum_{x=1}^{\infty }\lambda^{x}\exp\left(-\frac{1}{\ln\lambda }\left(\lambda^{x}-1\right)\right). \end{array}$$

The second moment is derived from the second derivative of $M_{X}\left(e^{s}\right)$:

$$\begin{array}{cc} E(X^{2}) & ={\left.\frac{d^{2}M_{X}\left(e^{s}\right)}{ds^{2}}\right|}_{s=0}=\sum_{x=1}^{\infty }\left(2x-1\right)\lambda^{x}\exp\left(-\frac{1}{\ln\lambda }\left(\lambda^{x}-1\right)\right). \end{array}$$

The third moment is obtained by differentiating $M_{X}\left(e^{s}\right)$ three times:

$$\begin{array}{cc} E(X^{3}) & ={\left.\frac{d^{3}M_{X}\left(e^{s}\right)}{ds^{3}}\right|}_{s=0}=\sum_{x=1}^{\infty }\left(3x^{2}-3x+1\right)\lambda^{x}\exp\left(-\frac{1}{\ln\lambda }\left(\lambda^{x}-1\right)\right). \end{array}$$

Finally, the fourth moment follows from the fourth derivative:

$$\begin{array}{cc} E(X^{4}) & ={\left.\frac{d^{4}M_{X}\left(e^{s}\right)}{ds^{4}}\right|}_{s=0}=\sum_{x=1}^{\infty }\left(4x^{3}-6x^{2}+4x-1\right)\lambda^{x}\exp\left(-\frac{1}{\ln\lambda }\left(\lambda^{x}-1\right)\right). \end{array}$$

Based on the derived moments, the variance, skewness, and kurtosis can be expressed as follows:

(19) $$\begin{array}{cc} \sigma^{2} & =E\left(X^{2}\right)-{\left[E\left(X\right)\right]}^{2}. \end{array}$$

(20) $$\begin{array}{cc} \mathrm{Skewness} & =\frac{E\left(X^{3}\right)-3E\left(X^{2}\right)E\left(X\right)+2{\left[E\left(X\right)\right]}^{3}}{{\left[\mathrm{Var}\left(X\right)\right]}^{3/2}}. \end{array}$$

(21) $$\begin{array}{cc} \mathrm{Kurtosis} & =\frac{E\left(X^{4}\right)-4E\left(X^{3}\right)E\left(X\right)+6E\left(X^{2}\right){\left[E\left(X\right)\right]}^{2}-3{\left[E\left(X\right)\right]}^{4}}{{\left[\mathrm{Var}\left(X\right)\right]}^{2}}. \end{array}$$

The dispersion index (DI) is described as the ratio of variance to the mean:

(22) $$\begin{array}{cc} \mathrm{DI} & =\frac{\mathrm{Variance}(X)}{E(X)}. \end{array}$$

- If DI < 1, the model is appropriate for under-dispersed data.
- If DI > 1, the model is appropriate for over-dispersed data.

Table 1 provides quantitative values for the mean, variance, dispersion index (DI), skewness, and kurtosis of the DsMuth distribution for various values of the model parameter λ. Based on Table 1, the following key observations can be observed:

1. As the parameter λ increases, the mean and variance of the DsMuth distribution gradually decline.
2. The dispersion index (DI) rises as λ increases, implying that the distribution becomes more dispersed with higher values of λ.
3. Skewness reduces as λ increases, implying that the distribution becomes less positively skewed for larger values of λ.
4. Kurtosis declines with increasing λ, meaning the distribution becomes less peaked (less leptokurtic) as λ grows.

**Table 1:** Descriptive statistics for the DsMuth distribution.

Measure	λ								
	1.1	1.2	1.3	1.4	1.6	1.8	2.0	2.2	2.4
Mean	0.575	0.568	0.562	0.556	0.545	0.535	0.526	0.517	0.510
Variance	0.772	0.671	0.597	0.540	0.456	0.398	0.356	0.324	0.300
Dispersion Index (DI)	0.744	0.846	0.941	1.030	1.194	1.342	1.476	1.596	1.700
Skewness	1.806	1.532	1.337	1.187	0.964	0.799	0.664	0.539	0.418
Kurtosis	13.14	11.239	10.041	9.176	7.916	6.954	6.110	5.246	4.330

### 3.2. Mean Residual Life (MRL)

The Mean Residual Life function is a crucial reliability characteristic used in modeling burn-in and maintenance policies. It provides the expected remaining lifetime given that a system or component has already survived until a specific point in time.

For a discrete random variable *X* with cumulative distribution function (CDF) F(x), the mean residual life function is described as:

$$\begin{array}{cc} \mathrm{MRL}=\varepsilon (i) & =E(X-i|X\geq i)\\ \; & =\frac{1}{1-F(i-1,\lambda )}\sum_{j=i+1}^{\infty }\left(1-F(j-1,\lambda )\right). \end{array}$$

Accordingly, the Mean Residual Life (MRL) function of the DsMuth model simplifies to:

$$\begin{array}{cc} \mathrm{MRL} & =\frac{1}{1-F(i-1,\lambda )}\sum_{j=i+1}^{\infty }\left(1-F(j-1,\lambda )\right). \end{array}$$

Further, substituting F(x) from the DsMuth distribution:

$$\begin{array}{cc} \mathrm{MRL} & =\frac{1}{\lambda^{i}\exp\left(-\frac{1}{\ln\lambda }\left(\lambda^{i}-1\right)\right)}\sum_{j=i+1}^{\infty }\lambda^{j}\exp\left(-\frac{1}{\ln\lambda }\left(\lambda^{j}-1\right)\right). \end{array}$$

## 4. Entropy

Entropy measures the fluctuation in the uncertainty in physical systems. The Shannon entropy is a well-known entropy measurement [35]. In this section, we obtain the discrete Shannon entropy of a random variable. The entropy H(X) of a discrete random variable *X*, with its probability mass function (pmf), is given by Equation (7):

$$H\left(X\right)=-\sum_{x=0}^{\infty }p\left(x\right)\log p\left(x\right)$$

The entropy of the DsMuth distribution can be computed using Mathcad software© var.15. The following formula is derived as follows:

(23) $$\begin{array}{cc} H(X) & =-\sum_{x=0}^{\infty }p\left(x\right)\log\left[\lambda^{x}\exp\left(-\frac{1}{\ln\lambda }\left(\lambda^{x}-1\right)\right)-\lambda^{\left(x+1\right)}\exp\left(-\frac{1}{\ln\lambda }\left(\lambda^{\left(x+1\right)}-1\right)\right)\right]\\ H(X) & =-\log\lambda \sum_{x=0}^{\infty }xp\left(x\right)+\frac{1}{\ln\lambda }\sum_{x=0}^{\infty }\left(\lambda^{x}-1\right)p\left(x\right)+\lambda \sum_{x=0}^{\infty }\left(1-\frac{1}{\ln\lambda }\left(\lambda^{\left(x+1\right)}-\lambda^{x}\right)\right)p\left(x\right). \end{array}$$

See Appendix A for the proof.

**Figure 6:**
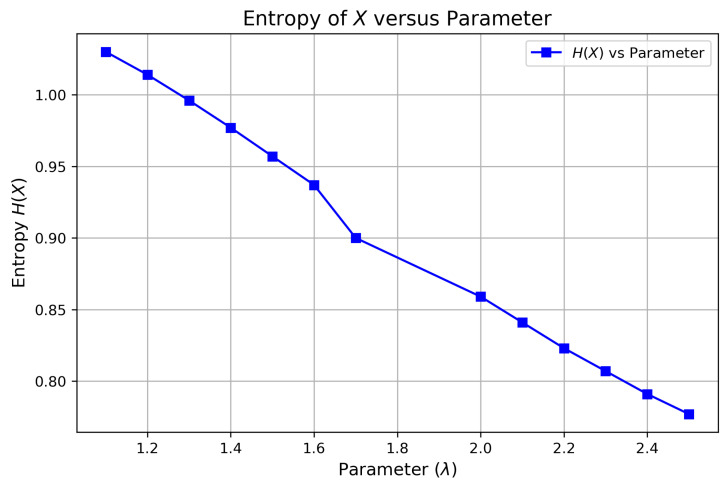
Entropyof H(X) versus parameter λ.

Table 2 provides numerical values for the entropy of a DsMuth distribution under various parameter choices, computed using Mathcad software© version 15. Additionally, Figure 6 illustrates the relationship between entropy, H(X), and the parameter values. Notably, the entropy function H(X) exhibits a monotonic decrease as the parameter λ varies within the interval λ∈(1,2.7].

## 5. Quantiles of a Discrete Random Variable

**Definition 1.**  *The point $y_{u}$ is referred to as the uth quantile of a discrete random variable Y if it satisfies the following conditions [36]:*

$$P\left(Y\leq y_{u}\right)\geq u\;\mathrm{and}\;P\left(Y\geq y_{u}\right)\geq 1-u.$$

*This can be represented in terms of the cumulative distribution function (CDF) F(Y) as:*

$$F\left(y_{u}-1\right)<u\leq F\left(y_{u}\right).$$

**Proposition 1.**  *The uth quantile of the DsMuth distribution can be obtained by:*

(24) $$y_{u}=\frac{1}{\alpha }\log\left\{-\alpha \;w\left[-\frac{1}{\alpha }\left(1-u\right)\exp\left(-\frac{1}{\alpha }\right)\right]\right\},\;\alpha \in \left(0,1\right]$$

*where w(x) is the Lambert function and ⌈·⌉ is the ceiling function, which rounds a given number up to the nearest integer. In other words, for any real number x, the ceiling function returns the smallest integer that is greater than or equal to x.*

*See Appendix B for the proof.*

**Quartiles of the DsMuth Distribution:** The quartiles of the DsMuth distribution can be calculated using the formulas below:

$$y_{0.25}=\frac{1}{\alpha }\log\left\{-\alpha w\left[-\frac{1}{\alpha }\left(1-0.25\right)\exp\left(-\frac{1}{\alpha }\right)\right]\right\}$$

$$y_{0.75}=\frac{1}{\alpha }\log\left\{-\alpha w\left[-\frac{1}{\alpha }\left(1-0.75\right)\exp\left(-\frac{1}{\alpha }\right)\right]\right\}$$

$$y_{0.5}=\frac{1}{\alpha }\log\left\{-\alpha w\left[-\frac{1}{\alpha }\left(1-0.5\right)\exp\left(-\frac{1}{\alpha }\right)\right]\right\}$$

A random integer can be generated from the proposed model using the inverse transformation method. This method converts a uniformly distributed random variable into a sample from the target distribution. Let *U* be a random number selected from a uniform distribution on (0,1). Then, a random number *Y* that follows the DsMuth(α) distribution is generated using Equation (24).

## 6. Estimation Methods

In this section, three different estimation techniques are employed to estimate the unknown parameter of the DsMuth distribution. The estimation techniques considered are the Maximum Likelihood Estimation (MLE) method, the Moments Method (MOM), and the Proportion Estimation (PE) method.

### 6.1. Maximum Likelihood Estimation

Assume a random sample $x_{1},x_{2},\cdots ,x_{n}$ from the DsMuth distribution. The log-likelihood function can be expressed as:

$$\begin{array}{cc} L & =\prod_{i=1}^{n}p\left(\bar{x};\alpha \right)\\ L & =\prod_{i=1}^{n}\left[\lambda^{x_{i}}\exp\left\{-\frac{1}{\ln\lambda }\left(\lambda^{x_{i}}-1\right)\right\}-\lambda^{\left(x_{i}+1\right)}\exp\left\{-\frac{1}{\ln\lambda }\left(\lambda^{\left(x_{i}+1\right)}-1\right)\right\}\right] \end{array}$$

Taking the logarithm:

(25) $$\begin{array}{cc} \log L & =\log\prod_{i=1}^{n}\left[\lambda^{x_{i}}\exp\left\{-\frac{1}{\ln\lambda }\left(\lambda^{x_{i}}-1\right)\right\}-\lambda^{\left(x_{i}+1\right)}\exp\left\{-\frac{1}{\ln\lambda }\left(\lambda^{\left(x_{i}+1\right)}-1\right)\right\}\right]\\ l & =\sum_{i=1}^{n}\log\left(\lambda^{x_{i}}\exp\left\{-\frac{1}{\ln\lambda }\left(\lambda^{x_{i}}-1\right)\right\}-\lambda^{\left(x_{i}+1\right)}\exp\left\{-\frac{1}{\ln\lambda }\left(\lambda^{\left(x_{i}+1\right)}-1\right)\right\}\right)\; \end{array}$$

By differentiating Equation (25) with respect to the parameter λ, we obtain the non-linear equation:

(26) $$\begin{array}{cc} \frac{dl}{d\lambda } & =\frac{d}{d\lambda }\sum_{i=1}^{n}\log\left(\lambda^{x_{i}}\exp\left\{-\frac{1}{\ln\lambda }\left(\lambda^{x_{i}}-1\right)\right\}-\lambda^{\left(x_{i}+1\right)}\exp\left\{-\frac{1}{\ln\lambda }\left(\lambda^{\left(x_{i}+1\right)}-1\right)\right\}\right)=0\\ \frac{dl}{d\alpha } & =\sum_{i=1}^{n}\frac{\left(\exp\left\{-\frac{1}{\ln\lambda }\left(\lambda^{x_{i}}-1\right)\right\}\left[x_{i}\lambda^{x_{i}-1}-\frac{x_{i}\lambda^{2x_{i}-1}}{\ln\lambda }+\frac{\lambda^{x_{i}}\left(\lambda^{x_{i}}-1\right)}{\lambda {\left(\ln\lambda \right)}^{2}}\right]\right)}{\lambda^{x_{i}}\exp\left\{-\frac{1}{\ln\lambda }\left(\lambda^{x_{i}}-1\right)\right\}-\lambda^{\left(x_{i}+1\right)}\exp\left\{-\frac{1}{\ln\lambda }\left(\lambda^{\left(x_{i}+1\right)}-1\right)\right\}}\\ \; & -\frac{\left(\exp\left\{-\frac{1}{\ln\lambda }\left(\lambda^{\left(x_{i}+1\right)}-1\right)\right\}\left[\left(x_{i}+1\right)\lambda^{x_{i}}-\frac{\left(x_{i}+1\right)\lambda^{2x_{i}-1}}{\ln\lambda }+\frac{\lambda^{\left(x_{i}+1\right)}\left(\lambda^{\left(x_{i}+1\right)}-1\right)}{\lambda {\left(\ln\lambda \right)}^{2}}\right]\right)}{\lambda^{x_{i}}\exp\left\{-\frac{1}{\ln\lambda }\left(\lambda^{x_{i}}-1\right)\right\}-\lambda^{\left(x_{i}+1\right)}\exp\left\{-\frac{1}{\ln\lambda }\left(\lambda^{\left(x_{i}+1\right)}-1\right)\right\}}=0\; \end{array}$$

The likelihood equation does not have a closed-form solution; however, the maximum likelihood estimate (MLE) of λ can be determined through numerical methods. By applying a numerical method to solve Equation (26), the estimators can be efficiently obtained using Mathcad software.

### 6.2. Method of Moment Estimation

Utilizing the method of moments (MOM) to estimate the parameter λ of the DsMuth distribution, we equate the sample mean to the theoretical population mean and solve for λ. This results in a non-linear equation that must be solved numerically. Thus, the estimator λ is obtained by solving the following equation with respect to λ:

(27) $$\sum_{x=1}^{\infty }x\lambda^{x}\exp\left[-\frac{1}{\ln\lambda }\left(\lambda^{x}-1\right)\right]=\frac{1}{n}\sum_{i=1}^{n}x_{i}\;$$

Since Equation (27) does not have a closed-form solution, numerical methods are required to determine its value. Therefore, a symbolic computation tool like Mathcad software should be used to solve the equation numerically, utilizing observed data points $x_{i},i=1,2,\cdots ,n$.

### 6.3. Method of Proportion Estimation

Assume a random sample $x_{1},x_{2},\ldots ,x_{n}$ drawn from the DsMuth distribution, which is characterized by a single parameter. Therefore, an indicator function is defined as follows:

(28) $$I\left(x_{i}\right)=\left\{\begin{array}{cc} 1, & \mathrm{if}\;x_{i}=0\\ 0, & \mathrm{otherwise} \end{array}\right.\;$$

Assume that $W=\sum_{i=1}^{n}I\left(x_{i}\right)$ represents the total count of zero observations in the sample. Using Equations (8) and (28), the probability of obtaining a zero observation is given by:

$$P\left(X\leq 0\right)=\frac{W}{n}$$

Based on this, the Proportion Estimation (PE) method is used to estimate the parameter λ. The estimate $\hat{\lambda }$ is obtained by solving the following equation:

$$1-\hat{\lambda }\exp\left\{-\frac{1}{\ln\hat{\lambda }}\left(\hat{\lambda }-1\right)\right\}-\frac{W}{n}=0.$$

Since $\frac{W}{n}$ is an unbiased and consistent empirical estimator of probability P(X≤0), it follows that the estimator $\hat{\lambda }$ also possesses consistency and biasedness in estimating λ. Further details on this can be found in [24,37].

## 7. Simulation Study

To verify the accuracy of the performance of the proposed estimators (MLE method, MME method, and PE method), we conducted a numerical simulation to assess the estimation based on the DsMuth model concerning the sample size *n*. The evaluation depends on the simulation procedure outlined below:

1. Generate 10,000 samples of size n=50,100,150,200,300,500 from DsMuth(λ), considering different values: DsMuth(1.1), DsMuth(1.2), DsMuth(1.7), DsMuth(1.8), DsMuth(2.2), and DsMuth(2.5), respectively. This simulation is performed using Mathcad Software.
2. A general expression to generate a random variable *X* from the DsMuth distribution is to first generate a value *Y* from the continuous distribution and then discretize this value to obtain *X*. Here, X = [Y], which represents the largest integer less than or equal to *Y* [38,39].
3. Compute the MLEs, MMEs, and PEs for the 10,000 samples, denoted as ${\hat{\lambda }}_{i}$ for i=1,2,⋯,10,000.
4. Compute the bias (B), mean square errors (MSEs), and mean relative error (MRE) of λ by employing three methods, specifically MLE, MME, and PE, using the following formulas:
  $$\mathrm{Bias}\left(\lambda \right)=\frac{1}{10,000}\sum_{i=1}^{10,000}\left({\hat{\lambda }}_{i}-\lambda \right)$$
  $$\mathrm{MSE}\left(\lambda \right)=\frac{1}{10,000}\sum_{i=1}^{10,000}{\left({\hat{\lambda }}_{i}-\lambda \right)}^{2}$$
  $$\mathrm{MRE}\left(\lambda \right)=\frac{1}{10,000}\sum_{i=1}^{10,000}\frac{|{\hat{\lambda }}_{i}-\lambda |}{\lambda }$$

The results obtained from the empirical analysis are presented in Table 3. The bias, MSE, and MRE of the parameter were computed using the Mathcad program with the three estimation methods. The following observations can be made from Table 3:

- **Improved Accuracy with Larger Sample Sizes:**
  The estimates of λ get closer to their true values as the sample size *n* increases across all estimation methods. This demonstrates the asymptotic property of the estimators, meaning they improve as more data become available.
- **Bias Reduction:**
  The bias of the parameter decreases toward **zero** as the sample size increases in all estimation methods. This indicates that the estimators are unbiased or asymptotically unbiased, ensuring greater accuracy in large samples.
- **Mean Squared Error (MSE) Decrease:**
  The MSE values decrease as *n* increases for all estimation methods. This confirms the consistency of the estimators, indicating that as more data is used, the estimates become more precise and less variable.
- **Mean Relative Error (MRE) Decrease:**
  The MRE also declines as *n* increases, further supporting the consistency of the estimators. This metric highlights how estimation errors become smaller in proportion to the true parameter value.
- **MLE as the Most Effective Method:**
  Among the three estimation techniques, Maximum Likelihood Estimation (MLE) performs best. It consistently provides estimates with the lowest bias, smallest MSE, and smallest MRE compared to the Method of Moments (MME) and Proportion Estimation (PE) methods. This suggests that MLE is the most efficient method for estimating λ in the DsMuth distribution.

**Table 3:** Simulation results of DsMuth distribution for several parameter values.

Parameter	n	AE			Bias			MSE			MRE		
		MLE	MME	PE	MLE	MME	PE	MLE	MME	PE	MLE	MME	PE
λ=1.1	50	1.054	0.773	0.931	−0.046	−0.327	−0.169	0.042	0.323	0.147	0.137	0.389	0.238
	100	1.054	0.806	1.011	−0.046	−0.294	−0.089	0.020	0.297	0.070	0.099	0.363	0.158
	150	1.075	0.795	1.027	−0.025	−0.305	−0.073	0.010	0.296	0.056	0.071	0.367	0.151
	200	1.079	0.881	1.022	−0.021	−0.219	−0.078	0.006	0.203	0.045	0.060	0.290	0.131
	300	1.091	0.905	1.057	−0.009	−0.195	−0.043	0.004	0.184	0.027	0.047	0.276	0.112
	500	1.098	0.938	1.079	−0.002	−0.162	−0.021	0.002	0.129	0.013	0.034	0.234	0.081
λ=1.2	50	1.153	0.873	1.028	−0.047	−0.327	−0.172	0.045	0.322	0.149	0.143	0.389	0.239
	100	1.167	0.899	1.087	−0.033	−0.301	−0.113	0.017	0.292	0.085	0.087	0.364	0.180
	150	1.180	0.903	1.123	−0.020	−0.297	−0.077	0.012	0.274	0.060	0.076	0.352	0.151
	200	1.184	0.959	1.138	−0.016	−0.241	−0.062	0.008	0.224	0.040	0.063	0.307	0.131
	300	1.189	0.976	1.158	−0.011	−0.224	−0.042	0.005	0.200	0.027	0.049	0.290	0.108
	500	1.194	1.014	1.172	−0.006	−0.186	−0.028	0.003	0.158	0.017	0.038	0.257	0.089
λ=1.7	50	1.646	1.655	1.753	−0.054	−0.045	0.053	0.112	0.439	0.155	0.131	0.374	0.217
	100	1.670	1.637	1.717	−0.030	−0.063	0.017	0.033	0.401	0.119	0.083	0.351	0.185
	150	1.684	1.609	1.727	−0.016	−0.091	0.027	0.022	0.375	0.106	0.071	0.335	0.173
	200	1.684	1.631	1.704	−0.016	−0.069	0.004	0.017	0.357	0.094	0.062	0.326	0.164
	300	1.703	1.653	1.697	−0.003	−0.047	−0.003	0.010	0.307	0.077	0.046	0.294	0.145
	500	1.698	1.655	1.698	−0.002	−0.045	−0.002	0.006	0.250	0.059	0.038	0.258	0.127
λ=1.8	50	1.713	1.771	1.998	−0.087	−0.029	0.198	0.199	0.427	0.195	0.141	0.341	0.193
	100	1.755	1.826	1.911	−0.045	0.026	0.111	0.046	0.387	0.119	0.089	0.317	0.156
	150	1.775	1.823	1.891	−0.025	0.023	0.091	0.026	0.364	0.095	0.069	0.301	0.141
	200	1.779	1.779	1.881	−0.021	−0.021	0.081	0.020	0.338	0.092	0.059	0.290	0.140
	300	1.784	1.799	1.852	−0.016	−0.001	0.052	0.013	0.272	0.063	0.049	0.248	0.114
	500	1.713	1.771	1.998	−0.009	−0.000	0.004	0.199	0.427	0.195	0.141	0.341	0.193
λ=2.2	50	1.947	2.356	2.673	−0.253	0.156	0.473	0.845	0.752	0.337	0.231	0.361	0.213
	100	2.132	2.231	2.627	−0.068	0.031	0.427	0.135	0.573	0.268	0.104	0.306	0.192
	150	2.160	2.233	2.591	−0.040	0.033	0.391	0.068	0.512	0.215	0.084	0.284	0.176
	200	2.180	2.227	2.557	−0.020	0.027	0.357	0.038	0.428	0.175	0.066	0.254	0.161
	300	2.183	2.222	2.547	−0.017	0.022	0.347	0.025	0.419	0.156	0.055	0.257	0.156
	500	2.185	2.170	2.507	−0.015	−0.030	0.307	0.014	0.296	0.113	0.041	0.208	0.138
λ=2.5	50	2.113	2.733	3.214	−0.387	0.233	0.714	1.049	0.704	0.630	0.269	0.298	0.290
	100	2.295	2.684	3.131	−0.205	0.184	0.631	0.413	0.596	0.470	0.155	0.269	0.256
	150	2.406	2.634	3.108	−0.094	0.134	0.608	0.201	0.487	0.425	0.110	0.243	0.247
	200	2.445	2.612	3.075	−0.055	0.112	0.575	0.081	0.405	0.371	0.084	0.223	0.234
	300	2.469	2.568	3.063	−0.031	0.068	0.563	0.041	0.341	0.352	0.063	0.203	0.229
	500	2.481	2.539	3.020	−0.019	0.039	0.520	0.020	0.254	0.286	0.045	0.174	0.211

## 8. Empirical Study

This section demonstrates the importance and superiority of the DsMuth distribution based on application to a real dataset. We compare the fits of the DsMuth distribution with several competitive distributions that have one or two parameters, including the discrete Rayleigh (DsR) [3], discrete Inverse-Rayleigh (DsIR) [33], discrete Lindley (DsLi) [10], Poisson (Poi) [40], discrete Poisson-Lindley (PoiLi) [41], discrete Lindley-Two Parameter (DLi-II) [14], discrete Linear Failure Rate (DLFR) [42], discrete Inverse Weibull (DIW) [7], and discrete Log-logistic (DLog-L) [15] distributions, which are listed in Table 4. The comparison of the fitted models is made using several criteria, including the negative maximum log-likelihood (-log-Lik.), Akaike information criterion (AIC), Bayesian information criterion (BIC), Hannan-Quinn information criterion (HQIC), and Chi-square ($\chi^{2}$) with its corresponding *p*-value.

The dataset includes the count of carious teeth among the four deciduous molars, with a sample size of 100. Detailed information about the dataset can be found in Krishna and Pundir [5,24]. The maximum likelihood estimates, standard errors (Std-er), confidence intervals (C.I), and goodness-of-fit measures for the dataset are provided in Table 5.

**Figure 7:**
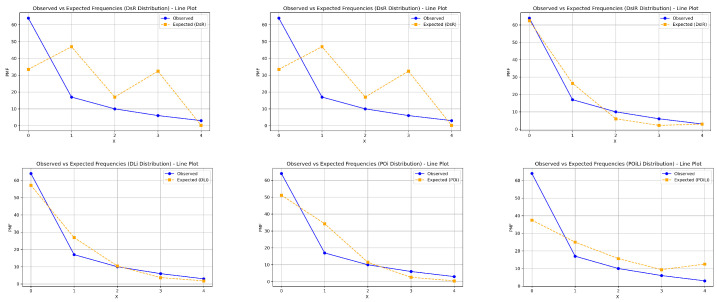
The probability mass functions (PMFs) fitted to the models with a single parameter for the dataset.

**Figure 8:**
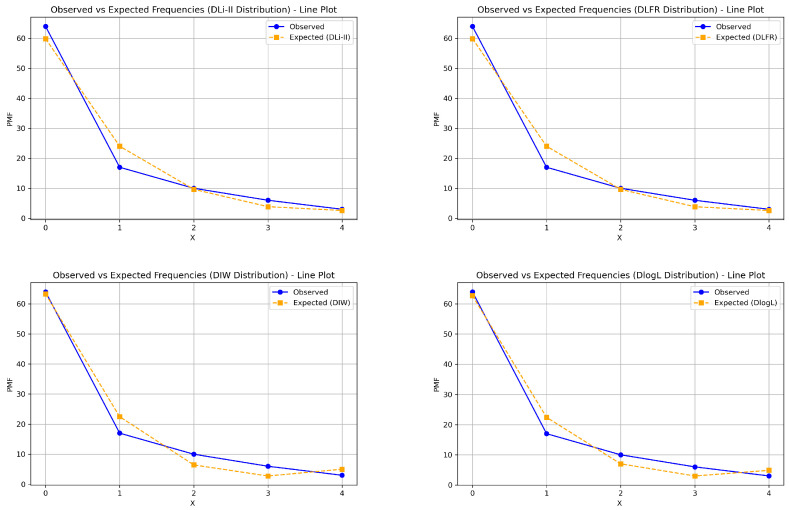
The probability mass functions (PMFs) fitted to the models with two parameters for the dataset.

According to Table 5, the DsMuth distribution proves to be the best choice for evaluating these data compared to other competitive distributions, as it has the lowest values for AIC, BIC, and HQIC, in addition to the highest *p*-value among all the tested distributions. Figure 7 and Figure 8 illustrate the fitted PMF plots for all tested distributions, further supporting the empirical results presented in Table 5.

## 9. Conclusions

This paper proposes a novel and flexible discrete distribution, known as the discrete Muth distribution, designed to effectively model count data that typically exhibit over-dispersion. The proposed distribution offers several advantageous properties that enhance its performance compared to various existing discrete distributions, especially in analyzing over-dispersed count data. Various structural characteristics of the DsMuth distribution are explored, including its mean, variance, skewness, kurtosis, probability generating function, moment generating function, mean residual life, moments, quantile function, and entropy. The model parameter is estimated to be employing different estimation techniques. Several simulation studies are conducted with various parameter settings, estimation techniques, and sample sizes. The results indicate that the model’s performance improves as the sample size increases, and it was observed that the maximum likelihood method is efficient for estimating the DsMuth parameters. Additionally, the flexibility and applicability of the proposed distribution are demonstrated through its application to a real dataset. The goodness of fit measures and graphical representations indicate that the DsMuth distribution is both effective and appealing when compared to other competing discrete distributions. Finally, the proposed distribution can be utilized as a replacement for existing distributions in the literature when modeling count data.

## Figures and Tables

**Table 2:** Entropy of *X* versus parameter λ.

Parameter λ	H(X)	Parameter λ	H(X)
1.1	1.030	1.7	0.900
1.2	1.014	2.0	0.859
1.3	0.996	2.1	0.841
1.4	0.977	2.2	0.823
1.5	0.957	2.3	0.807
1.6	0.937	2.4	0.791

**Table 4:** The competitive distributions of the DsMuth distribution with their pmfs.

Distribution	Abbreviation	Author	Pmf
Discrete-Rayleigh	DsR	[3]	$p\left(x\right)=e^{-\frac{x^{2}}{2\lambda^{2}}}-e^{-\frac{{\left(x+1\right)}^{2}}{2\lambda^{2}}}$
Discrete Inverse-Rayleigh	DsIR	[33]	$p\left(x\right)=e^{-\frac{\lambda }{{\left(1+x\right)}^{2}}}-e^{-\frac{\lambda }{x^{2}}}$
Discrete Lindley	DsLi	[11]	$p\left(x\right)=\frac{\lambda^{x}\left[\lambda \log\lambda +\left(1-\lambda \right)\left(1-\log\lambda^{x+1}\right)\right]}{1-\log\lambda }$
Poisson	Poi	[40]	$p\left(x\right)=\frac{e^{-\lambda }\lambda^{x}}{x!}$
Discrete Poisson-Lindley	PoiLi	[41]	$p\left(x\right)=\frac{\theta^{2}\left(\theta +2+x\right)}{\left(\theta +1\right)x^{3}}$
Discrete Lindley-Two Parameter	DLi-II	[14]	$p\left(x\right)=\frac{{\left(1+p\right)}^{2}\left(1+Bx\right)p^{x}}{1+p(B-1)}$
Discrete linear failure rate	DLFR	[42]	$p\left(x\right)=\frac{\lambda +\beta (x-1)}{1+\beta (x-1)}$
Discrete Inverse Weibull	DIW	[7]	$p\left(x\right)=\left(\frac{\beta }{\lambda }\right){\left(\frac{x}{\lambda }\right)}^{-\beta -1}\exp\left(-{\left(\frac{x}{\lambda }\right)}^{-\beta }\right)$
Discrete Log-logistic	DLog-L	[15]	$p\left(x\right)=\frac{1}{\left[1+{\left(\frac{x+1}{\lambda }\right)}^{-\delta }\right]}-\frac{1}{\left[1+{\left(\frac{x}{\lambda }\right)}^{-\delta }\right]}$

**Table 5:** The MLE, Std-er, C.I., and goodness of fit measures for distributions with one or two parameters for the dataset.

X	Obs. Freq.	One-Parameter							Two-Parameter		
		DsMuth	DR	DIR	DLi	Poi	PoiLi	DLi-II	DLFR	DIW	DLLogL
0	64	63.21	33.50	62.50	57.13	51.17	37.50	59.88	59.90	63.30	62.73
1	17	23.25	46.94	26.41	26.88	34.28	25.00	24.02	24.01	22.48	22.42
2	10	8.556	17.01	5.99	10.45	11.49	15.63	9.64	9.63	6.44	7.01
3	6	3.147	2.39	2.19	3.71	2.57	9.38	3.87	3.86	2.76	2.98
≥4	3	1.583	0.16	2.91	1.83	0.49	12.49	2.59	2.60	5.02	4.86
**Total**		100	100	100	100	100	100	100	100	100	100
MLE		1.001	0.665	0.625	0.274	0.670	1.998	0.401	0.401	0.633	0.745
Std-er		0.01	0.029	0.049	0.029	0.082	0.263	0.269	0.056	0.049	0.101
L.C.I		1.000	0.608	0.529	0.217	0.509	1.481	0.000	0.291	0.537	0.546
U.C.I		1.019	0.722	0.721	0.331	0.831	2.514	0.928	0.511	0.729	0.944
$\chi^{2}$		3.934	66.07	9.056	6.638	23.65	30.889	3.347	3.340	3.503	2.783
DF		2	2	2	2	2	2	1	1	1	1
*p*-value		0.140	<0.001	0.011	0.036	<0.001	<0.001	0.067	0.068	0.061	0.095
−log Lik.		112.8	205.3	118.4	113.1	120.3	112.1	112.47	112.47	116.27	115.47
AIC		227.7	412.6	238.9	230.8	242.7	226.2	228.95	228.94	236.55	234.94
BIC		230.3	415.2	241.5	230.8	245.3	228.8	234.16	234.15	241.76	240.15
HQIC		228.7	413.7	240.0	229.2	243.8	227.3	231.06	231.05	238.66	237.04

## Data Availability

Stated in the text.

## Appendix A

The discrete Shannon entropy H(X) of a discrete random variable *X* with probability mass function (PMF) is given by Equation (7):

$$\begin{array}{cc} H(X) & =-\sum_{x=0}^{\infty }p\left(x\right)\log p\left(x\right).\\ H(X) & =-\sum_{x=0}^{\infty }p\left(x\right)\log\left[\lambda^{x}\exp\left(-\frac{1}{\ln\lambda }\left(\lambda^{x}-1\right)\right)-\lambda^{\left(x+1\right)}\exp\left(-\frac{1}{\ln\lambda }\left(\lambda^{\left(x+1\right)}-1\right)\right)\right]. \end{array}$$

For small values where 0<b<a, we use:

$$\log\left[a-b\right]\approx \log a+\log\left(1-b/a\right).$$

For small *z*, the logarithmic expansion is:

log(1−z)≈−z.

This leads to the following. Using the logarithm of subtraction property (approximation for small differences), we define:

$$\begin{array}{cc} p_{1}\left(x\right) & =\lambda^{x}\exp\left(-\frac{1}{\ln\lambda }\left(\lambda^{x}-1\right)\right),\\ p_{2}\left(x\right) & =\lambda^{\left(x+1\right)}\exp\left(-\frac{1}{\ln\lambda }\left(\lambda^{\left(x+1\right)}-1\right)\right). \end{array}$$

Thus, entropy simplifies as:

$$H\left(X\right)=-\sum_{x=0}^{\infty }p\left(x\right)\log\left[p_{1}\left(x\right)-p_{2}\left(x\right)\right].$$

Using the logarithmic identity approximation:

$$\log\left[p_{1}\left(x\right)-p_{2}\left(x\right)\right]\approx \log p_{1}\left(x\right)+\log\left(1-\frac{p_{2}\left(x\right)}{p_{1}\left(x\right)}\right).$$

$$\log\left[p_{1}\left(x\right)-p_{2}\left(x\right)\right]\approx \log\left(\lambda^{x}\exp\left(-\frac{1}{\ln\lambda }\left(\lambda^{x}-1\right)\right)\right)+\log\left(1-\frac{\lambda^{\left(x+1\right)}\exp\left(-\frac{1}{\ln\lambda }\left(\lambda^{\left(x+1\right)}-1\right)\right)}{\lambda^{x}\exp\left(-\frac{1}{\ln\lambda }\left(\lambda^{x}-1\right)\right)}\right).$$

Simplifying further:

$$\log p\left(x\right)=\log\left[p_{1}\left(x\right)-p_{2}\left(x\right)\right]\approx x\log\lambda -\frac{1}{\ln\lambda }\left(\lambda^{x}-1\right)+\log\left(1-\lambda \exp\left(-\frac{1}{\ln\lambda }\left(\lambda^{\left(x+1\right)}-\lambda^{x}\right)\right)\right).$$

Since:

log(1−z)≈−z,

we obtain:

$$H\left(X\right)=-\sum_{x=0}^{\infty }p\left(x\right)\left[x\log\lambda -\frac{1}{\ln\lambda }\left(\lambda^{x}-1\right)-\lambda \exp\left(-\frac{1}{\ln\lambda }\left(\lambda^{\left(x+1\right)}-\lambda^{x}\right)\right)\right].$$

For small $\lambda^{\left(x+1\right)}-\lambda^{x}$, using Taylor expansion:

exp(−z)≈1−z,

Thus:

$$\exp\left(-\frac{1}{\ln\lambda }\left(\lambda^{\left(x+1\right)}-\lambda^{x}\right)\right)\approx 1-\frac{1}{\ln\lambda }\left(\lambda^{\left(x+1\right)}-\lambda^{x}\right).$$

Final entropy expression:

$$H\left(X\right)=-\log\lambda \sum_{x=0}^{\infty }xp\left(x\right)+\frac{1}{\ln\lambda }\sum_{x=0}^{\infty }\left(\lambda^{x}-1\right)p\left(x\right)+\lambda \sum_{x=0}^{\infty }\left(1-\frac{1}{\ln\lambda }\left(\lambda^{\left(x+1\right)}-\lambda^{x}\right)\right)p\left(x\right).$$

## Appendix B

**Proof of Proposition A1.** The proof involves the following algebraic operations:

$$P\left(Y\leq y_{u}\right)=F\left(y_{u}\right)\geq u$$

$$1-\exp\left[\alpha \left(y_{u}+1\right)-\frac{1}{\alpha }\left(e^{\alpha (y_{u}+1)}-1\right)\right]\geq u$$

$$\exp\left[\alpha \left(y_{u}+1\right)-\frac{1}{\alpha }\left(e^{\alpha (y_{u}+1)}-1\right)\right]\leq 1-u$$

By setting $\Phi =e^{\alpha (y_{u}+1)}$, we obtain:

$$\Phi =e^{\alpha (y_{u}+1)}$$

$$\Phi \exp\left(\frac{-1}{\alpha }\Phi \right)\exp\left(\frac{1}{\alpha }\right)\leq 1-u$$

$$\Phi \exp\left(\frac{-1}{\alpha }\Phi \right)\leq \left(1-u\right)\exp\left(-\frac{1}{\alpha }\right)$$

Multiplying by $-\frac{1}{\alpha }$:

$$\left[-\frac{1}{\alpha }\right]\Phi \exp\left(\frac{-1}{\alpha }\Phi \right)\geq \left[-\frac{1}{\alpha }\right]\left(1-u\right)\exp\left(-\frac{1}{\alpha }\right)$$

Here, w(z) represents the Lambert function, which satisfies:

$$w\left(z\right)e^{w(z)}=z.$$

Thus, we obtain:

$$\left[-\frac{1}{\alpha }\right]\Phi \geq w\left(-\frac{1}{\alpha }\left(1-u\right)\exp\left(-\frac{1}{\alpha }\right)\right)$$

$$\Phi \leq -\alpha w\left(-\frac{1}{\alpha }\left(1-u\right)\exp\left(-\frac{1}{\alpha }\right)\right)$$

$$e^{\alpha (y_{u}+1)}\leq -\alpha w\left(-\frac{1}{\alpha }\left(1-u\right)\exp\left(-\frac{1}{\alpha }\right)\right)$$

(A1) $$y_{u}\leq \frac{1}{\alpha }\log\left\{-\alpha w\left(-\frac{1}{\alpha }\left(1-u\right)\exp\left(-\frac{1}{\alpha }\right)\right)\right\}-1\;$$

Similarly, solving for $P\left(Y\geq y_{u}\right)=1-P\left(Y\leq y_{u}-1\right)\geq 1-u$ gives:

$$1-\left\{1-\left[e^{\alpha \left(y_{u}\right)-\frac{1}{\alpha }\left(e^{\alpha (y_{u})}-1\right)}\right]\right\}\geq 1-u$$

$$e^{\alpha \left(y_{u}\right)-\frac{1}{\alpha }\left(e^{\alpha (y_{u})}-1\right)}\geq 1-u$$

Setting $\Phi =e^{\alpha (y_{u})}$, we obtain:

$$\Phi \exp\left(\frac{-1}{\alpha }\Phi \right)\exp\left(\frac{1}{\alpha }\right)\geq 1-u$$

$$\Phi \exp\left(\frac{-1}{\alpha }\Phi \right)\geq \left(1-u\right)\exp\left(-\frac{1}{\alpha }\right)$$

Multiplying by $-\frac{1}{\alpha }$:

$$\left[-\frac{1}{\alpha }\right]\Phi \exp\left(\frac{-1}{\alpha }\Phi \right)\leq \left[-\frac{1}{\alpha }\right]\left(1-u\right)\exp\left(-\frac{1}{\alpha }\right)$$

Using the Lambert function:

$$\left[-\frac{1}{\alpha }\right]\Phi \leq w\left(-\frac{1}{\alpha }\left(1-u\right)\exp\left(-\frac{1}{\alpha }\right)\right)$$

$$\Phi \geq -\alpha w\left(-\frac{1}{\alpha }\left(1-u\right)\exp\left(-\frac{1}{\alpha }\right)\right)$$

(A2) $$y_{u}\geq \frac{1}{\alpha }\log\left\{-\alpha w\left(-\frac{1}{\alpha }\left(1-u\right)\exp\left(-\frac{1}{\alpha }\right)\right)\right\}\;$$

Combining $\left[A_{1}\right]$ and $\left[A_{2}\right]$, we get:

$$\frac{1}{\alpha }\log\left\{-\alpha w\left(-\frac{1}{\alpha }\left(1-u\right)\exp\left(-\frac{1}{\alpha }\right)\right)\right\}<y_{u}\leq \frac{1}{\alpha }\log\left\{-\alpha w\left(-\frac{1}{\alpha }\left(1-u\right)\exp\left(-\frac{1}{\alpha }\right)\right)\right\}-1$$

Therefore, $y_{u}$ is an integer given by:

$$y_{u}=\left\lceil \frac{1}{\alpha }\log\left\{-\alpha w\left(-\frac{1}{\alpha }\left(1-u\right)\exp\left(-\frac{1}{\alpha }\right)\right)\right\}\right\rceil .$$

□
